# The Malagarasi River Does Not Form an Absolute Barrier to Chimpanzee Movement in Western Tanzania

**DOI:** 10.1371/journal.pone.0058965

**Published:** 2013-03-11

**Authors:** Alex K. Piel, Fiona A. Stewart, Lilian Pintea, Yingying Li, Miguel A. Ramirez, Dorothy E. Loy, Patricia A. Crystal, Gerald H. Learn, Leslie A. Knapp, Paul M. Sharp, Beatrice H. Hahn

**Affiliations:** 1 Department of Anthropology, University of California San Diego, San Diego, California, United States of America; 2 Division of Biological Anthropology, University of Cambridge, Cambridge, United Kingdom; 3 The Jane Goodall Institute, Arlington, Virginia, United States of America; 4 Departments of Medicine and Microbiology, University of Pennsylvania, Philadelphia, United States of America; 5 Institute of Evolutionary Biology and Centre for Immunity, Infection and Evolution, University of Edinburgh, Edinburgh, United Kingdom; Texas A&M University, United States of America

## Abstract

The Malagarasi River has long been thought to be a barrier to chimpanzee movements in western Tanzania. This potential geographic boundary could affect chimpanzee ranging behavior, population connectivity and pathogen transmission, and thus has implications for conservation strategies and government policy. Indeed, based on mitochondrial DNA sequence comparisons it was recently argued that chimpanzees from communities to the north and to the south of the Malagarasi are surprisingly distantly related, suggesting that the river prevents gene flow. To investigate this, we conducted a survey along the Malagarasi River. We found a ford comprised of rocks that researchers could cross on foot. On a trail leading to this ford, we collected 13 fresh fecal samples containing chimpanzee DNA, two of which tested positive for SIVcpz. We also found chimpanzee feces within the riverbed. Taken together, this evidence suggests that the Malagarasi River is not an absolute barrier to chimpanzee movements and communities from the areas to the north and south should be considered a single population. These results have important consequences for our understanding of gene flow, disease dynamics and conservation management.

## Introduction

Large mammals are capable of long-range dispersal provided suitable, contiguous habitat exists [1]–[4] but the influence of large rivers, specifically, in preventing movement of individuals has been documented in several species [5]–[7]. Geographic barriers are suggested to play a key role in the formation of species by preventing gene flow, resulting in the accumulation of genetic differences and ultimately divergence of populations into separate species [8]. A similar process of disrupted gene flow due to such geographic barriers is suggested to influence population structure within species. Such processes have been documented in each of the great apes: orangutans [9], gorillas [10], bonobos [11], and chimpanzees [12].

Across Africa, much of the suitable chimpanzee habitat has disappeared since the early 1990s [13]. The loss, degradation, and fragmentation of suitable habitats can have a similar effect to natural barriers, thereby reducing the potential for dispersal and movement of such large-bodied mammals. Thus, understanding current, as well as historical, patterns of gene flow between and within populations has important implications for chimpanzee conservation and will aid in determining specific areas to preserve population connectivity in increasingly human-utilized landscapes.

Chimpanzees (*Pan troglodytes*) are widely distributed across equatorial Africa, and the formation of four sub-species (*P.t. verus, P.t. ellioti, P.t. troglodytes, P.t. schweinfurthii*) is, in part, attributed to large river systems that historically (and currently) prevent gene flow [12]. Chimpanzees have a fluid social structure where sub-groups of individuals fission and fuse flexibly in response to ecological (fruit availability and distribution) or social (estrus females) factors. They live in communities characterized by linear male dominance hierarchies and territorial defense, often showing lethal inter-group aggression [14]. Most females (50–90%) disperse from their natal group at sexual maturity to reproduce in another community [15], and in eastern chimpanzees especially, male migration rarely, if ever, occurs [16]. Given this female dispersal bias, mtDNA has commonly been used to reconstruct population history, both on large [17], [18] and small [19] scales. In Guinea (Conakry), for example, Shimada and colleagues [19] used this technique to examine population connectivity between the increasingly isolated Bossou chimpanzees and those from the Nimba community, which live in contiguous habitat stretching into Cote d'Ivoire. The authors found very few shared mtDNA variants across the two sites, suggesting minimal gene flow. However, the variants identified within each area are scattered throughout the subspecies, suggesting any genetic separation between these areas is recent. Thus, mtDNA markers can reveal both current and historic connectivity between chimpanzee communities.

**Figure 1:**
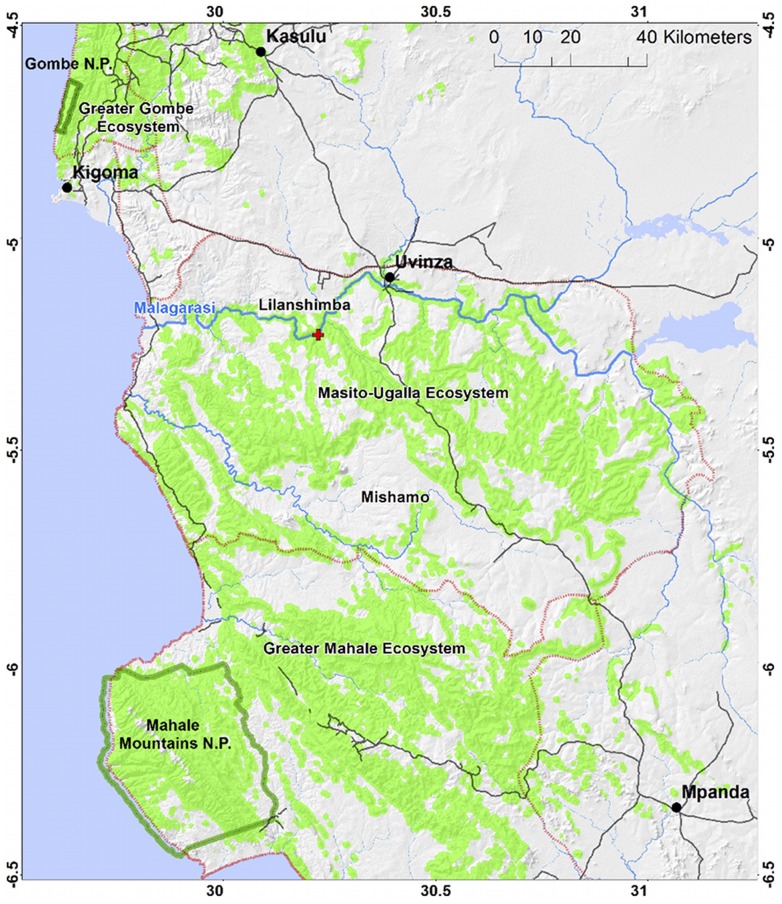
Greater Gombe, Masito-Ugalla and Greater Mahale Ecosystem (outlined in red dots). The predicted distribution of suitable chimpanzee nesting habitat was developed using the Mahalanobis distance model [50], updated with 2007–2012 nest data and 2007 vegetation cover from Landsat TM satellite imagery (green). A red cross indicates the location of the crossing point over the Malagarasi River (blue).

The range of the eastern chimpanzee (*P.t. schweinfurthii*) spans the Democratic Republic of Congo north of the Congo River, Uganda, Rwanda, Burundi and Tanzania [12]. In Tanzania, surveys have estimated as many as 2800 individuals, 75% of which live outside of national parks [20]. Most of Tanzania's chimpanzees (∼2600) inhabit the Greater Mahale Ecosystem (GME, Figure 1), with only 110 estimated to remain in the Greater Gombe Ecosystem (GGE) containing Gombe National Park in the north, and <80 in Loasi in the south of the country. The combination of forest-clearing, especially for agriculture, threatens important chimpanzee habitat, and has nearly isolated Gombe chimpanzees specifically [21]. Despite these threats, the presence of small clusters of chimpanzee nests in fragmented forest patches suggests there may still be some movement within the GGE landscape between Gombe and the GME.

The GME is a 20,000 km^2^ area bordered by the Ugalla River to the east and the Malagarasi to the north, and includes Mahale Mountains National Park to the west (Figure 1) [22]. The extent to which these rivers prevent chimpanzee movement is debated. There have been previous efforts both by air (L. Pintea, unpublished data) and by water (H. Ogawa, unpublished data), to investigate the impact of the Malagarasi as a biogeographical boundary, but direct evidence concerning whether chimpanzees are able to cross the river is lacking.

The Malagarasi River begins near the Burundian border, is 475 km long, and has the largest watershed of all the rivers flowing into Lake Tanganyika. In the Miocene, the lower Malagarasi was the headwater of the Congo River; today, Lake Tanganyka serves as a holding basin for the Malagarasi, before its waters meet the Lukuga River [23] and eventually the Atlantic Ocean [24]. The upper Malagarasi, on the other hand, was historically part of the Nile drainage, flowing towards Lake Victoria until later tectonic activity reversed its direction [23]. The river is more than 100 m wide for long stretches and in some parts crocodile can be seen. Moreover, the river banks are bordered by extensive stretches of miombo woodland plateau – a topographical feature that savanna chimpanzees rarely use for feeding or nesting (unpublished data). Given that chimpanzees are not known to swim [25], the extent to which this river is a boundary to chimpanzee movement has important implications. For example, such a boundary could impact chimpanzee ranging behavior [26], population connectivity, and pathogen transmission [27], [28], and thus has implications for conservation strategies and government policy.

**Figure 2:**
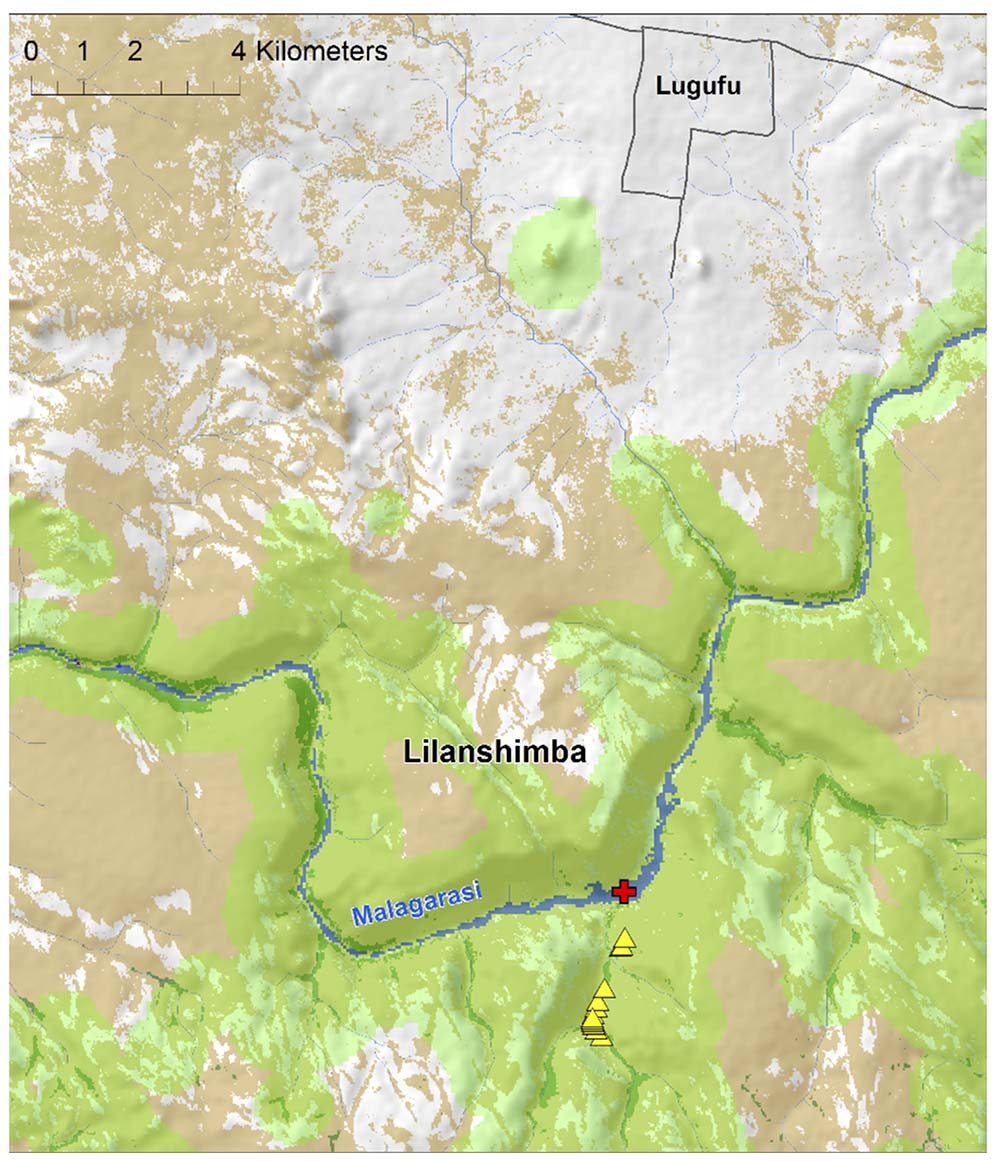
Location of the Malagarasi River ford. The location of the crossing point (red cross) is shown in relation to forest (dark green), woodland (brown) and water (blue). The chimpanzee distribution model is shown in light green, with land cover classes extracted from 2007 Landsat TM satellite image and draped over a digital elevation map (shaded relief from 90-m SRTM - Shuttle Radar Topography Mission – data). Yellow triangles depict the location of chimpanzee fecal samples.

North of the river, but outside of Gombe, there is minimal evidence of chimpanzee presence. In the Lilanshimba area [29]–[31], just north of the river (Figure 2), Ogawa et al. [31] proposed that two chimpanzee communities, totaling 32–45 individuals, may have existed prior to the establishment of the Lugufu refugee settlement in the 1990s. Recent evidence suggests that some of these chimpanzees may have survived to the present day, despite more than 100,000 refugees from the Democratic Republic of Congo inhabiting this settlement for 15 years until 2009. If the Malagarasi River were to form an absolute barrier, chimpanzees remaining in Gombe (∼110) and those in Lilanshimba (∼45) would be isolated from the larger GME population and would likely not represent a viable sub-population. In fact, Inoue et al. [32] have recently proposed that the Malagarasi River represents a barrier to chimpanzee gene flow [29], [30]. After examining 138 fecal samples, they found no shared mitochondrial DNA (mtDNA) haplotypes between chimpanzees from Gombe (6 samples), about 90 km to the north of the Malagarasi River, and those from the Greater Mahale area (including Masito-Ugalla) south of the river. In fact, chimpanzees from south of the river were more similar, in mtDNA sequences, to individuals from Loasi (Lwazi), about 200 km further south near the southern end of Lake Tanganyika, and the authors argued that chimpanzees had historically reached the Greater Mahale area from the south [32].

In the current study, we integrate three independent data sets to re-evaluate the extent to which the Malagarasi is a boundary to chimpanzee movements. We use (1) previously published and new genetic data from chimpanzee fecal samples, (2) SIVcpz distribution, and (3) observations of the river itself. Specifically, we report here the discovery of a ford in the Malagarasi River that is sufficiently shallow for chimpanzees to readily cross. Furthermore, we found chimpanzee feces on the approach to and in the vicinity of this ford. Here we describe this evidence and discuss the implications of such a crossing place for current and historical gene flow between Masito-Ugalla and Greater Gombe Ecosystems.

## Materials and Methods

### Ethics Statement

Permission to survey and collect noninvasive fecal samples from wild chimpanzees was provided to AKP (No. 2011-247-NA) and FAS (No. 2011-248-NA) by the national institutions TAWIRI (Tanzanian Wildlife Research Institute) and COSTECH (Commission for Science and Technology), whilst District governments in both Kigoma and Mpanda authorized the project and specifically AKP and FAS to survey on General (District) Land. Additionally, we met all ethical and legal requirements established by the American Society of Primatologists (ASP) for work on wild primates.

### Study sites and sample collection

We established a camp 19 km south of the Malagarasi River (at −5.40, 30.20) on 29 July 2012, in the middle of the dry season, with a subsequent camp (6 km from the river, at −5.28, 30.22) established on 3 August 2012. Camps were basic, selected for their proximity to water, away from any human habitation, and with the goal of minimizing disturbance to chimpanzees.

Research teams used a combination of QuickBird, SPOT and Landsat satellite imagery and predictive distribution models of potential chimpanzee nesting habitat [33] to decide where to target daily reconnaissance walks, with the goal of investigating forest patches lining the river systems and miombo woodland slopes for the presence of chimpanzees. These vegetation types are known to be preferred for chimpanzee nesting from previous surveys and research [34], [35]. We recorded data on all chimpanzee nests, vocalizations, and fecal samples encountered, including GPS coordinates and apparent age (nest age was recorded according to state of decay [35], [36], from age 1 to 4 as follows: (1) leaves green and nest structure intact; (2) some leaves brown, but nest structure intact; (3) nest rotting and structure disintegrating; and (4) only the frame and <5% of leaves remaining. Fecal samples were aged as follows: from 1–3 with 1  =  fresh, within 12 hours, 2  =  recent, within 1–2 days, and 3  =  old, dried and >2 days. Whenever possible recent signs of chimpanzees were tracked by following trails in order to maximize fresh fecal sample collection success. Only fresh fecal samples were collected, usually from beneath chimpanzee night nests, or from following chimpanzee trails, when encountered during reconnaissance walks. Fecal samples were collected on 7 August 2012, and were preserved in an equal volume of RNA*later* (Life Technologies) [37]–[39]. Tubes were labeled with a sample number and GPS coordinates. Because of a lack of refrigeration, at small satellite camp locations, samples were kept at ambient temperature for up to one week before longer term storage at base-camps at 4°C in a DC refrigerator (Model number ARB, 47L) powered by a portable power system (4 Lucas 75Ah sealed batteries, and 2 Yingli 60 W crystalline solar panels). Samples were stored at 4°C for several weeks, but in some instances several months, before they were shipped to the Hahn laboratory, University of Pennsylvania, and stored at −20°C.

Individual identification. All fecal samples were subjected to mitochondrial DNA analysis to confirm their species origin. Briefly, a 498 bp fragment of the mitochondrial D-loop region was amplified from fecal DNA, sequenced directly (using one primer which yielded 446 bp of sequence) and grouped into different haplotypes [38]–[40]. The evolutionary relationships of these haplotypes to each other and to a reference set of other available Tanzanian chimpanzee D-loop sequences [32] from the database were then determined by phylogenetic analysis. A neighbor-joining tree [41] was constructed based on the region where all sequences overlapped using CLUSTALW version 2.11 [42] with distances corrected for multiple substitutions [43]. Bootstrap confidence values [44] are based on 1000 replicates.

To identify the number of sampled individuals, fecal samples were genotyped at eight autosomal microsatellite loci [27], [37], [45]–[47] with amplification products sized on an automated sequencer (Applied Biosystems). Sex was determined by amplifying a 218 bp fragment of the sex-linked amelogenin gene that contains a 6 bp insertion in the Y, but not the X chromosome [48].

SIVcpz detection. All fecal samples were also screened for the presence of HIV-1 cross-reactive antibodies by enhanced chemiluminescent Western immunoblot analysis modified for RNA*later* preserved specimens [37]–[39]. Sample integrity was examined using an IgG control. Western blot positive samples were tested for the presence of SIV nucleic acids by reverse-transcription polymerase chain reaction (RT-PCR) amplification using SIVcpz specific *pol* primers [49].

## Results

Forty-two chimpanzee nests were observed on 7 August 2012. Among these, only one was Age1, six were Age2, twenty-nine were Age3, and six were Age4. Following evergreen forest patches and pristine chimpanzee habitat, we identified a well-used trail that led from a forested mountain area approximately 5 km south of the Malagarasi River directly to the river itself (Figure 2). On this trail, we identified sixteen fresh chimpanzee fecal samples, indicating the recent presence of chimpanzees in very close proximity to the southern bank of the Malagarasi River.

**Figure 3:**
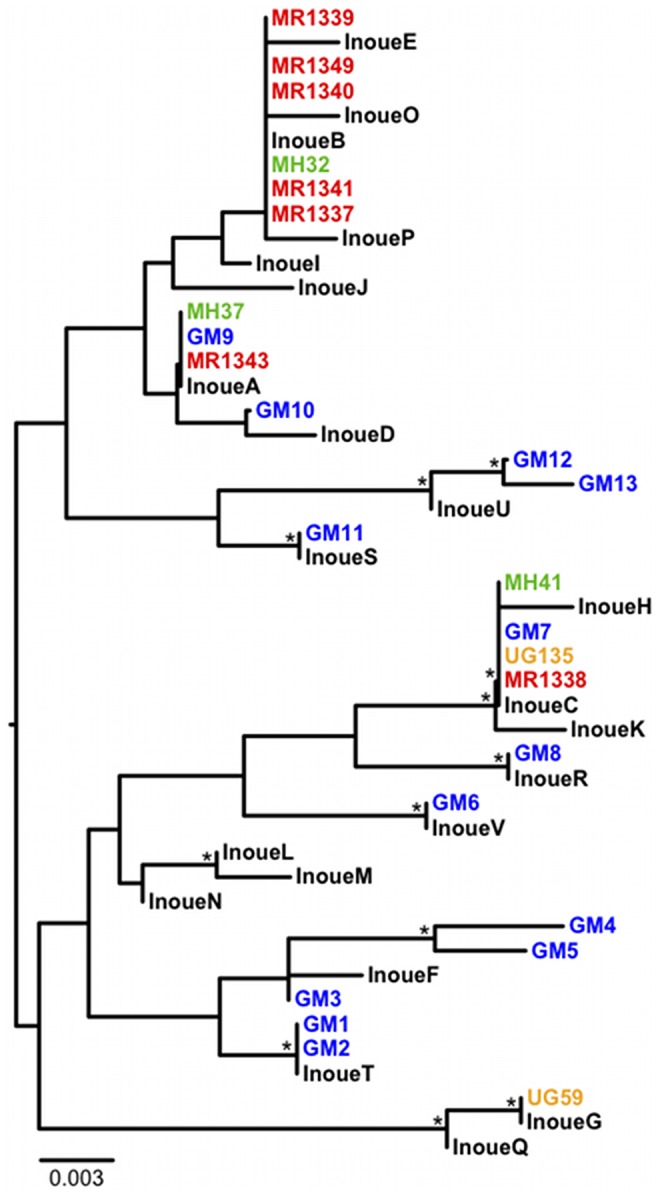
Mitochondrial genetic diversity in chimpanzees from Tanzania. Mitochondrial DNA (D loop) sequences from chimpanzees sampled on the southern bank of the Malagarasi River (MR, red) are shown in relation to previously identified chimpanzee mitochondrial haplotypes from the Gombe Stream National Park (GM, blue) [45], Mahale Mountains National Park (MH, green) [40] and Ugalla (UG, orange) [27]. Haplotypes reported by Inoue and colleagues [32] are shown in black. The tree was constructed using the neighbor joining method (asterisks denote bootstrap support values above 70%). The scale bars represents 0.003 substitutions per site.

To confirm the origin of these fecal samples, we extracted DNA from all 16 specimens and amplified the D loop region of the mtDNA genome. Direct amplicon sequencing identified three samples that comprised fecal mixtures, which were excluded from further analysis. The remaining 13 samples yielded three different mtDNA haplotypes (Table 1), all of which clustered with other eastern chimpanzee (*Pan troglodytes schweinfurthii*) sequences (Figure 3). All three of the sequences had previously been found in samples from chimpanzees in Mahale National Park (MH32, MH37 and MH40), including one haplotype that has also been identified in chimpanzees in Gombe National Park (GM7). Thus, we compared results from the current samples to all known published haplotypes of eastern chimpanzees, creating a comprehensive dataset that allowed us to make inferences on gene flow.

**Table 1:** SIVcpz infection in chimpanzees sampled near the Malagarasi river.

No	Chimp ID^a^	Sample code	Collection date	Sex^b^	mtDNA haplotype^c^	Microsatellite loci^d^								SIVcpz fecal WB^e^	SIVcpz fecal vRNA
						D18s536	D4s243	D10s676	D9s922	D2s1326	D2s1333	D4s1627	D9s905		
1	Ch-1	MR1338	Aug-7-12	M	GM7	137/173	192/227	174/182	286/302	232/260	314/318	221/225	295/295	neg	
2		MR1347	Aug-7-12	M	GM7	137/173	192/227	174/182	286/302	232/260	314/318	221/225	295/295	neg	
3		MR1348	Aug-7-12	M	GM7	137/173	192/227	174/182	286/302	232/260	314/318	221/225	295/295	neg	
4		MR1350	Aug-7-12	M	GM7	137/173	192/227	174/182	286/302	232/260	314/318	221/225	295/295	neg	
****5	**Ch-2**	**MR1349**	**Aug-7-12**	**M**	**MH32**	**137/173**	**200/227**	**174/174**	**280/302**	**228/252**	**318/334**	**221/233**	**295/295**	**pos**	**neg**
6	Ch-3	MR 1355	Aug-7-12	M	MH32	133/173	192/231	174/194	286/298	248/248	?/318	221/233	?/295	neg	
7		MR1340	Aug-7-12	n/a	MH32	133/173	?/231	?/?	?/?	248/248	302/?	221/233	291/?	neg	
8	Ch-4	MR1339	Aug-7-12	M	MH32	137/157	192/231	174/182	286/290	228/252	306/318	221/233	283/287	neg	
9		MR1342	Aug-7-12	M	MH32	137/157	192/231	174/182	286/290	228/252	306/318	221/233	283/287	neg	
10		MR1344	Aug-7-12	M	MH32	137/157	192/231	174/182	286/290	228/252	306/318	221/233	283/287	neg	
11	**Ch-5**	**MR1343**	**Aug-7-12**	**F**	**MH37**	**137/173**	**223/227**	**174/174**	**290/290**	**228/252**	**314/318**	**221/229**	**287/287**	**pos**	**neg**
12	Ch-6	MR1337	Aug-7-12	M	MH32	133/137	192/231	178/182	290/302	248/252	318/334	221/233	287/295	neg	
13	Ch-7	MR1341	Aug-7-12	F	MH32	137/149	215/227	178/182	290/302	228/260	314/322	221/221	295/295	neg	

a SIVcpz infected chimpanzees are indicated by boldface.

b F, female; M, male; n/a, not available.

c GM7, MH37 and MH32 haplotypes have previously been reported [40].

d ?/?, Partial genotype due to sample degradation.

e WB, Western blot; pos, positive; neg, negative.

To determine the number of individual chimpanzees, we subjected all mtDNA positive fecal samples to microsatellite analyses (Table 1). One of these (MR1340) failed to yield a complete genotype, due to partial sample degradation. Since MR1340 shared the same mtDNA haplotype and three STR loci with sample MR1355, we conservatively classified it to represent the same individual (Table 1). The remaining 11 samples represented six additional individuals, indicating a total of seven sampled chimpanzees, including five males and two females (Table 1). Five of these individuals shared the same mtDNA haplotype (MH32). None of these samples exhibited the same genotype as individuals sampled in other areas of the Masito-Ugalla ecosystem ([27]; unpublished).

**Figure 4:**
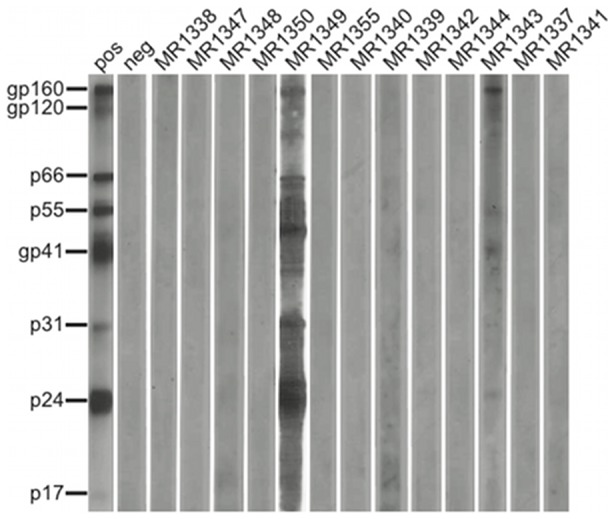
Evidence of SIVcpz infection on the southern bank of the Malagarasi River. Chimpanzee fecal samples were tested for HIV/SIV specific antibodies using an enhanced chemiluminescent Western blot approach and HIV-1 antigen containing strips. Samples from individuals (Ch-2 and Ch-5; Table 1) reacted with envelope, integrase and/or core proteins, indicating SIVcpz infection. Molecular weights of HIV-1 specific proteins are indicated. The banding pattern of plasma from an HIV-1 infected patient (pos at a 1∶10,000 dilution) and an uninfected human control (neg) are shown.

To determine whether any of the Malagarasi chimpanzees were SIVcpz infected, we tested all genotyped fecal samples for the presence of antibodies using a highly sensitive and specific Western blot approach [37]. Interestingly, two of the sampled individuals, one male and one female, exhibited clear evidence of SIVcpz infection (Figure 4). Sample MR1349 reacted strongly with multiple HIV-1 proteins, including the viral envelope (gp160), integrase (p31) and core (p24) proteins. Sample MR1343 was also Western blot positive, albeit only weakly, yielding diagnostic gp160 and p24 bands. Unfortunately, multiple attempts to amplify SIVcpz sequences from the two antibody positive samples failed, most likely due to partial sample degradation.

**Figure 5:**
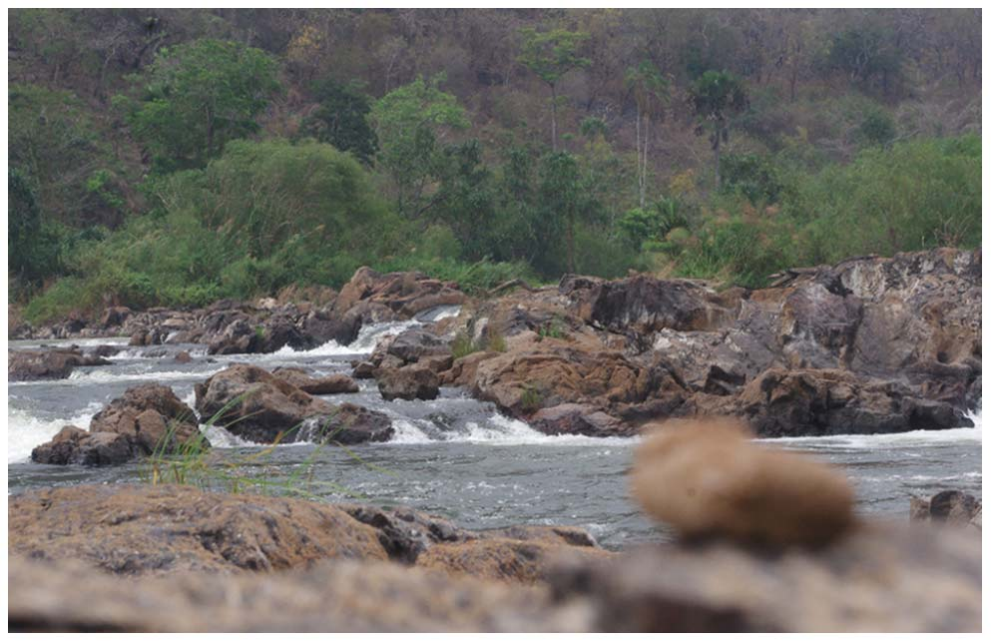
Photograph of location where researchers, and presumably chimpanzees, can cross the river. In the foreground is a dried chimpanzee fecal sample.

Finally, we found a natural shallow ford in the Malagarasi River, where investigators were able to cross the river in its entirety on foot, jumping from rock to rock. On examination of the area, we found two fecal samples on rocks in the riverbed (Figure 5). These samples were not collected because they had dried up, and thus were not considered suitable for molecular analyses. However, careful inspection revealed that they each had the characteristic shape, size, and smell of chimpanzee feces. Taken together, these findings strongly suggest that chimpanzees approach and are able to cross the river, at least during the dry season.

## Discussion

Habitat loss from the conversion of forest and woodlands to agricultural land is one of the most significant threats to chimpanzee survival in Tanzania. Establishing the location of natural boundaries for Tanzania's chimpanzees has implications for how we interpret current and historical population connectivity and advise policy-makers on human land-use strategies that may have significant effects on chimpanzee habitat. The Malagarasi River has been proposed as a major biogeographical boundary that has impeded the movement of chimpanzees, as well as other mammals [29], [32]. Such barriers have been well documented to have significant impacts on gene flow and overall genetic diversity in all three other apes [9]–[11], and thus the consequences for further chimpanzee fragmentation in Tanzania are significant for population viability as well as conservation strategy.

Inoue et al.′s [32] recent study proposed two important features to the population genetic landscape in western Tanzania. By comparing mtDNA haplotypes from 138 samples between chimpanzees from Gombe and those from the GME south of the Malagarasi, they found no shared haplotypes. They drew two conclusions: First, that the Malagarasi River limits gene flow between these two regions. Second, and equally important, that the southern sampled areas (Wansisi Hilla, Mahale, Kalobwe, and Ugalla-Masito) represent a single population. Our results dispute both of these claims with important implications for conservation (see below). It is only by integrating comprehensive and independent data sets from (1) published haplotypes, (2) SIVcpz distribution, and (3) ground truthing that we can thoroughly address this important question of chimpanzee fragmentation.

First, Inoue et al. [32] had very few samples from Gombe. Comparing sequences from our more extensive sampling of the Gombe chimpanzee communities [28], [37], we find that mtDNA haplotypes A, C and R found by Inoue et al. [32] in the GME (but not at Gombe) are identical to haplotypes GM9, GM7 and GM8 from Gombe chimpanzees (Figure 3). Thus, the communities to the north and south of the Malagarasi are not as genetically distinct as reported. Second, we provide circumstantial evidence that the Malagarasi River does not, at least seasonally, present the physical barrier to individual chimpanzee movement that would prevent gene flow. We surveyed the southern bank of the river in the ‘Masito’ region for shallow areas that could be crossed without wading or swimming through deep water (Figure 5). Following a possible chimpanzee trail, we found one such ford. We also found recent chimpanzee nests and fresh knuckle prints leading right up to the riverbed, as well as 13 fecal samples confirmed to be of chimpanzee origin. Moreover, we found dried fecal samples of likely chimpanzee origin on rocks within the riverbed. Thus it appears that chimpanzees approach the river at this location and, at least in the dry season, would be able to cross it as researchers did.

The idea that chimpanzees can cross the Malagarasi is further supported by earlier work describing the molecular epidemiology of simian immunodeficiency virus (SIVcpz). In that study, Rudicell et al. [27] reported that chimpanzees from the Issa Valley community (in Ugalla, south of the river) share some mtDNA haplotypes with those from Gombe National Park. At the time, it was unknown whether this reflected recent gene flow across the river or more distant common ancestry. However, the results presented here would suggest the former. Furthermore, we also found closely related strains of SIVcpz infecting chimpanzees in Gombe and in the Issa Valley, respectively about 90 km north and 50 km south of the Malagarasi River [27], raising the possibility that these locations are epidemiologically linked. Together, these results strongly suggest that, in contrast to Inoue et al. [32], there is gene flow and also disease transmission across the Malagarasi River.

If chimpanzees indeed frequently use the ford that we identified as a natural bridge, the implications are threefold. First, it suggests that the Greater Gombe and Masito-Ugalla regions might have historically had some gene flow, even if limited by season or rainfall, with chimpanzees on both sides being connected to a greater extent than previously thought [32]. The possibility that these two systems are connected is important for conservationists and researchers alike, who seek to prevent additional chimpanzee communities from becoming isolated due to human disturbance. If the river is a barrier to chimpanzee movement, Lilanshimba chimpanzees [29], [31] are unlikely to be a viable population, especially unless habitat connectivity can be restored to Gombe. If, however, the river is not an absolute barrier, then those chimpanzees are part of the GME and we should mobilize resources to protecting remaining habitat in the area, especially now that the nearby Lugufu Refugee Camp has been closed and there is a dramatic reduction in human pressure on the area.

Second, since the discovery of SIVcpz in chimpanzees from Gombe [38] and more recently, Ugalla [27], researchers have suspected a transmission path across the Malagarasi. The connectivity reported here may now explain how SIVcpz spread to chimpanzees south of the Malagarasi [27] and potentially be a threat to the other GME chimpanzees. Inoue et al. [32] argue that close genetic distance across the GME suggests this area represents a single population. This is inconsistent with recent SIVcpz results that reveal no positive infections in over 400 samples collected across the southern GME (unpublished data). Thus, whilst it remains possible that a recent introduction of SIVcpz to the region from the north explains its absence in the south, a more parsimonious explanation is that the GME is actually not a single population. More investigation is necessary to confirm where potential barriers may exist within the GME.

Finally, our data serve as a reminder that thorough investigation of potential ‘barriers’ is critical to confirm their actual role in chimpanzee population health, movements, and potential disease transmission. Since the mid 1960s, researchers have described the Malagarasi River as a boundary to chimpanzee movement, before thoroughly investigating possible routes across it. As a result, local conservation organizations designated two entirely different ‘Ecosystems’ (Greater Mahale to the south; Greater Gombe to the north). Our results suggest that this nomenclature is misleading. Further, currently a second large river has come under similar scrutiny. The Lugufu River runs parallel to the Malagarasi, south <10 km near the Mkuyu village forest where chimpanzees have been observed. Knowing whether this is a permanent or merely seasonal barrier has important implications for conservation planning, especially regarding population viability assessment.

Of course, the finding of chimpanzee fecal samples in close proximity to and within the Malagarasi riverbed does not provide conclusive evidence of connectivity across the river. However, Ogawa et al.′s [29], [31] description of chimpanzees living within the Lilanshimba area to the north of the river suggests that, rather than faced with imminent extermination, chimpanzees there may be able to move southward, escaping expanding human settlement, into more remote areas. Moreover, the natural bridge that we describe could have been used extensively in the past, thus explaining evidence of shared mtDNA haplotypes and closely related viral genotypes on both sides of the river. Although recent work has begun to shed light on the genetic relatedness of chimpanzees in western Tanzania [32], more comprehensive field and genetic surveys are needed in Lilanshimba, Loasi, and the GME, to provide more detailed knowledge of western Tanzanian chimpanzee gene flow, ecology and disease susceptibility.
