# Correction: The Malagarasi River Does Not Form an Absolute Barrier to Chimpanzee Movement in Western Tanzania

**DOI:** 10.1371/journal.pone.0182723

**Published:** 2017-08-01

**Authors:** Alex K. Piel, Fiona A. Stewart, Lilian Pintea, Yingying Li, Miguel A. Ramirez, Dorothy E. Loy, Patricia A. Crystal, Gerald H. Learn, Leslie A. Knapp, Paul M. Sharp, Beatrice H. Hahn

The authors would like to correct Fig 4. During the preparation of Fig 4, the negative strips for lanes “neg” and MR1344, as well as lanes MR1355 and MR1337 were mistakenly duplicated. Additionally, the labels for lanes MR1349 and MR1343 were switched.

**Fig 4:**
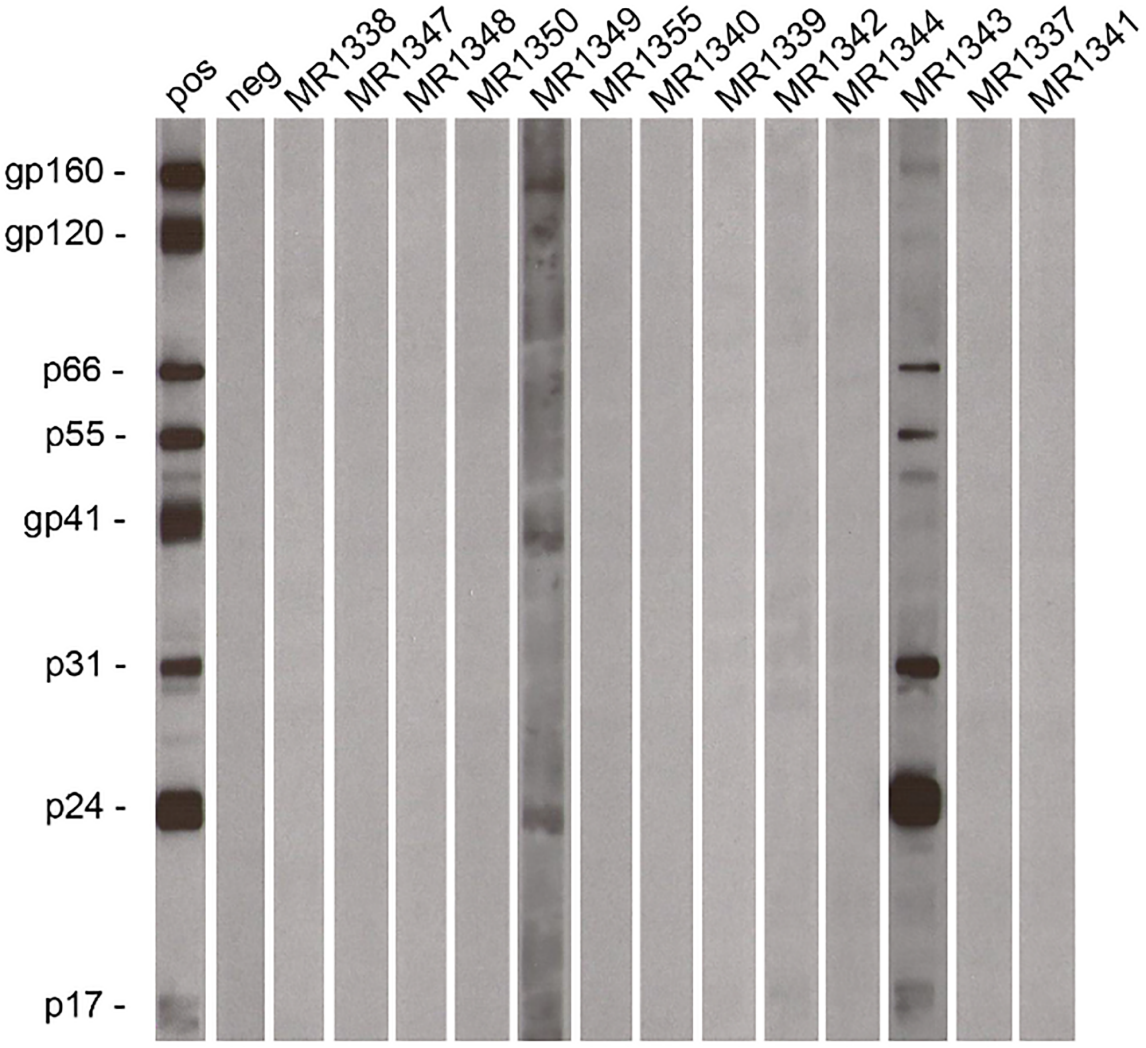
Evidence of SIVcpz infection on the southern bank of the Malagarasi River. Chimpanzee fecal samples were tested for HIV/SIV specific antibodies using an enhanced chemiluminescent Western blot approach and HIV-1 antigen containing strips. Samples from individuals (Ch-2 and Ch-5; Table 1) reacted with envelope, integrase and/or core proteins, indicating SIVcpz infection. Molecular weights of HIV-1 specific proteins are indicated. The banding pattern of plasma from an HIV-1 infected subject (pos at a 1:1,000,000 dilution) and an uninfected human control (neg) are shown.

The authors have repeated the Western blot analysis of the entire set of chimpanzee fecal samples reported in the study. Specifically, they went back to the original fecal specimens (which were stored at -80^°^C), took another 1.5 ml aliquot from each sample, and then processed these aliquots to generate fecal extracts for enhanced chemiluminescent Western immunoblot analysis as originally described. The authors have provided a corrected version of Fig 4 here.

The authors state that the data as they were reported in the original paper are correct, that the repeat Western blots confirm the presence of SIVcpz antibodies in samples MR1349 and MR1343, and not in any of the other eleven chimpanzee fecal samples, but that in contrast to previous observations sample MR1349 is only weakly positive, while sample MR1343 is strongly positive. The authors have provided raw, uncropped blots from the new analysis as a Supporting Information file.

## Supporting information

S1 File Uncropped blots from the new analysis. Four different X-ray exposures are shown for the same set of Western blot strips (panel A, 10 sec; panel B, 15 sec; panel C, 20 sec; panel D, 30 sec), with panel C used to generate the new Figure 4. Three additional controls (not included in Figure 4) are shown: Hu pos 10^−7^, plasma from the same HIV-1 infected subject shown as the positive control (pos) in Figure 4, but used at a 1:10,000,000 dilution; Chimp pos 10^−6^, plasma from a captive chimpanzee experimentally infected with HIV-1 used at a 1:1,000,000 dilution (additional positive control); GM4522, fecal extract from an uninfected chimpanzee (additional negative control).
(PDF)
