# Supplementary material for: Correction: The Malagarasi River Does Not Form an Absolute Barrier to Chimpanzee Movement in Western Tanzania
Source: PLoS One. 2017 Aug 1;12(8):e0182723. doi: 10.1371/journal.pone.0182723 (PMC5538667; doi:10.1371/journal.pone.0182723)
Supplement: S1 File — Four different X-ray exposures are shown for the same set of Western blot strips (panel A, 10 sec; panel B, 15 sec; panel C, 20 sec; panel D, 30 sec), with panel C used to generate the new Figure 4. Three additional controls (not included in Figure 4) are shown: Hu pos 10−7, plasma from the same HIV-1 infected subject shown as the positive control (pos) in Figure 4, but used at a 1:10,000,000 dilution; Chimp pos 10−6, plasma from a captive chimpanzee experimentally infected with HIV-1 used at a 1:1,000,000 dilution (additional positive control); GM4522, fecal extract from an uninfected chimpanzee (additional negative control). (PDF) [file pone.0182723.s001.pdf]

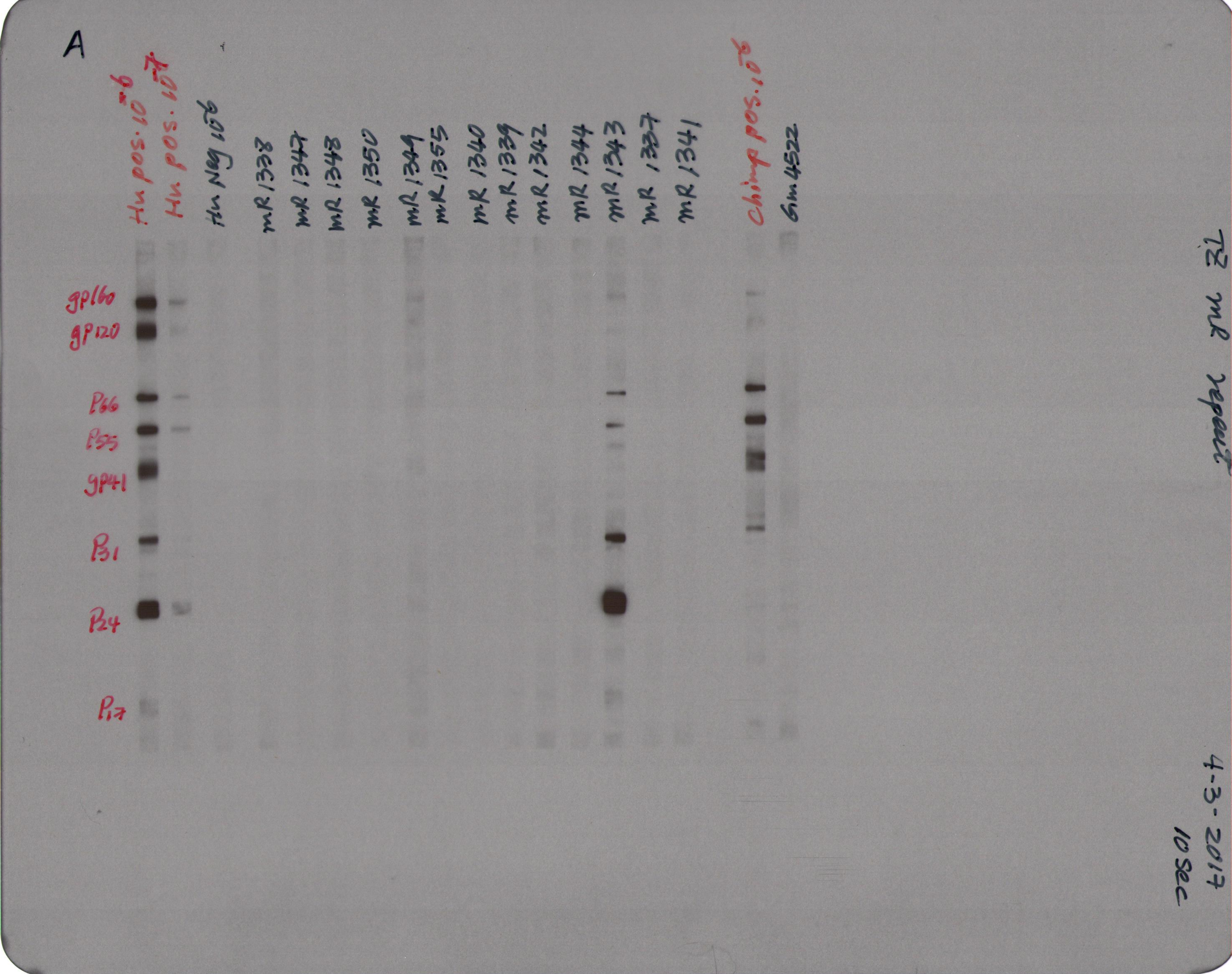
A
Hu pos. 10-6
Hu pos. 10-7
Hu Neg 10-2
mR 1338
mR 1347
mR 1348
mR 1350
mR 1349
mR 1355
mR 1340
mR 1339
mR 1342
mR 1344
mR 1343
mR 1337
mR 1341
Chimp pos. 10-2
Gm 4522
gp160
gp120
P66
P55
gp41
P31
P24
P17
TZ mR repeat
4-3-2017
10 sec

B

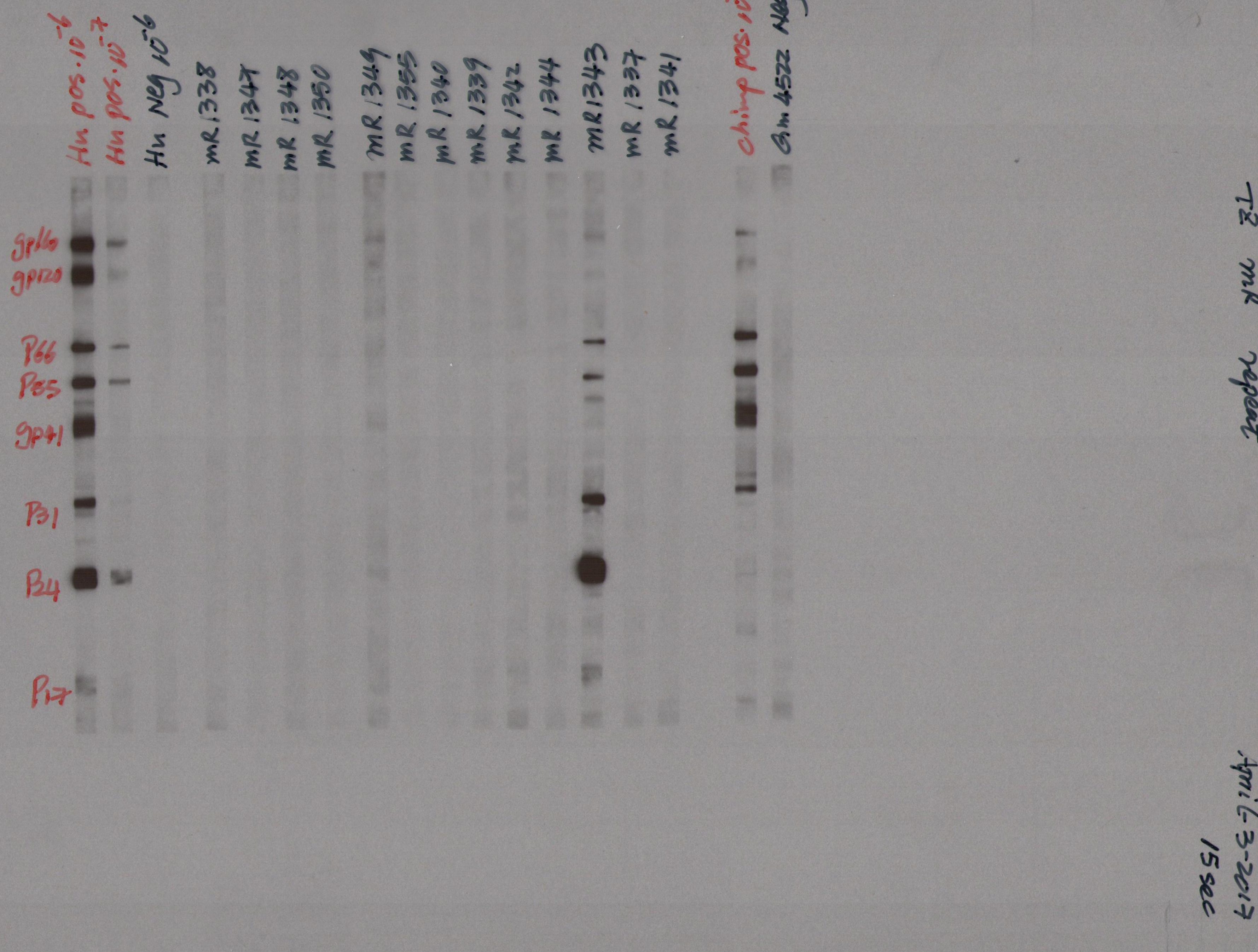

Hu pos·10-6
Hu pos·10-7
Hu Neg 10-6
mR1338
mR 1347
mR 1348
mR 1350
mR 1349
mR 1355
mR 1340
mR 1339
mR 1342
mR 1344
mR1343
mR 1337
mR 1341
chimp pos·10-6
Gm 4522 Neg
gp160
gp120
P66
P55
gp41
P31
P24
P17
mR repeat
April-3-2017
15 sec

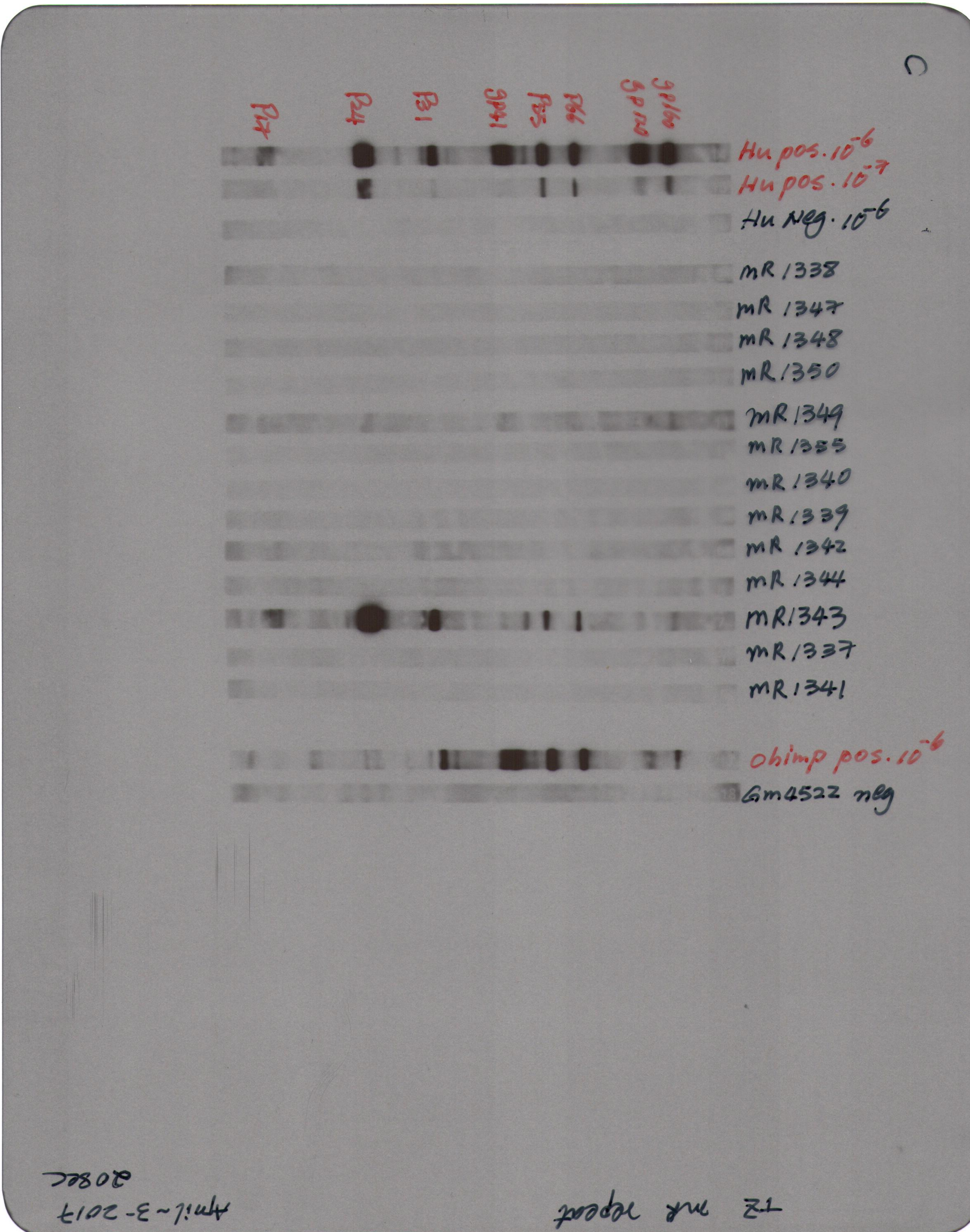
C
p17
p24
p31
gp41
p55
p66
gp120
gp160
Hu pos. 10-6
Hu pos. 10-7
Hu neg. 10-6
MR 1338
MR 1347
MR 1348
MR 1350
MR 1349
MR 1355
MR 1340
MR 1339
MR 1342
MR 1344
MR 1343
MR 1337
MR 1341
chimp pos. 10-6
GM4522 neg
TZ MR repeat
April-3-2017
20 sec

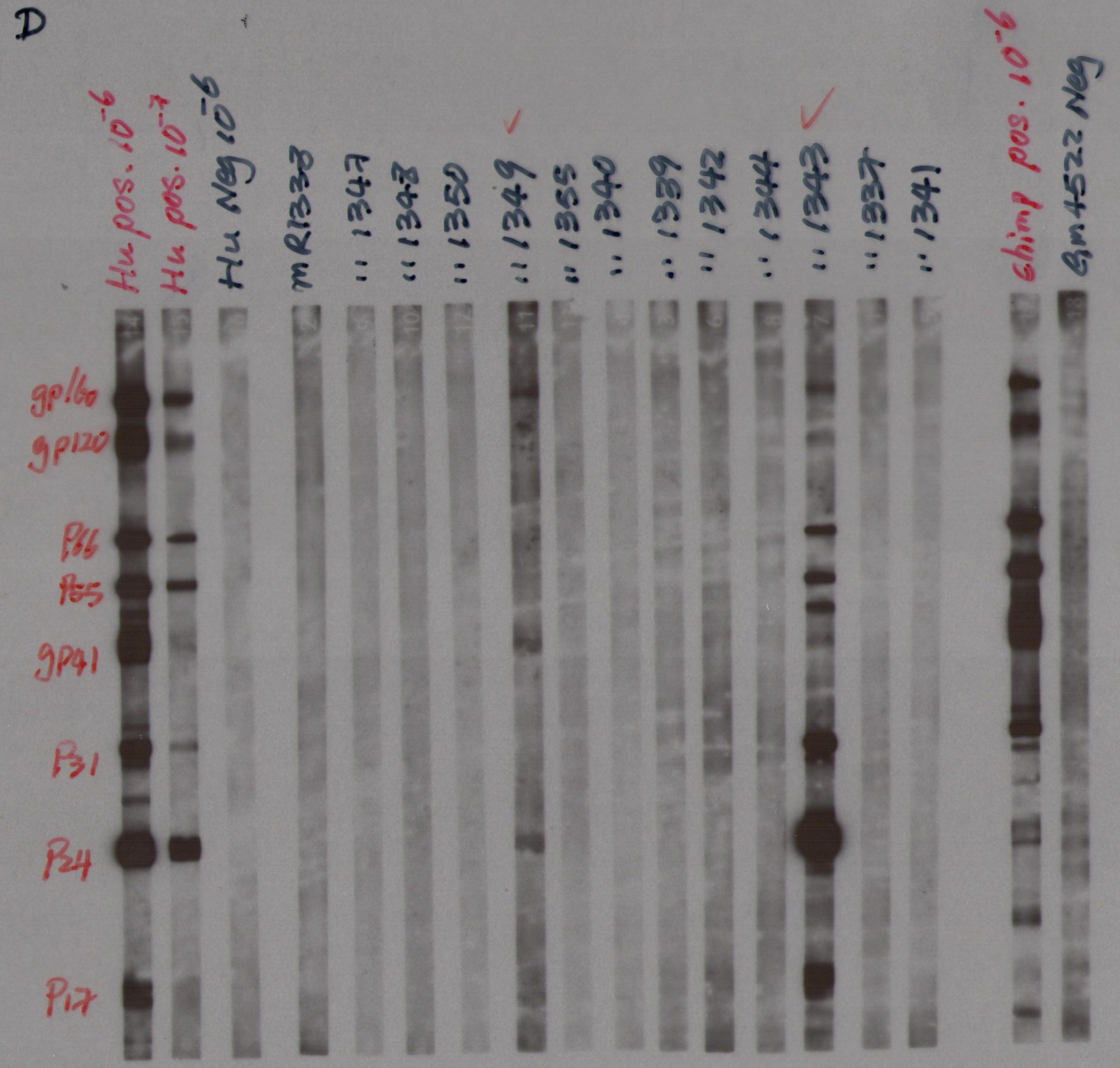

TZ MR repeat

4-3-2017
30sec
